# Macrophage-regulatory T cell immune circuits shape the pre-metastatic niche

**DOI:** 10.1016/j.cpt.2026.04.003

**Published:** 2026-05-08

**Authors:** Qamar Abuhassan, Ghaleb Oriquat, Waleed K. Abdulsahib, R. Roopashree, Priya Priyadarshini Nayak, J. Bethanney Janney, Vipasha Sharma, Xushvakt Umedov

**Affiliations:** a Department of Pharmaceutics and Pharmaceutical Technology, School of Pharmacy, University of Jordan, Amman 11942, Jordan; b Faculty of Allied Medical Sciences, Hourani Center for Applied Scientific Research, Al-Ahliyya Amman University, Amman 11942, Jordan; c Department of Pharmacology and Toxicology, College of Pharmacy, Al Farahidi University, Baghdad 10070, Iraq; d Department of Chemistry and Biochemistry, School of Sciences, JAIN (Deemed to be University), Bangalore, Karnataka 560027, India; e Department of Medical Oncology, IMS and SUM Hospital, Siksha ‘O’ Anusandhan (Deemed to be University), Bhubaneswar, Odisha 751003, India; f Department of Biomedical Engineering, Sathyabama Institute of Science and Technology, Chennai, Tamil Nadu 600119, India; g Department of Biotechnology, University Institute of Biotechnology, Chandigarh University, Mohali, Punjab 140413, India; h Department of Surgical Diseases No. 2, Samarkand State Medical University, Samarkand 140100, Uzbekistan

**Keywords:** Extracellular vesicles, Macrophage, Pre-metastatic niche, Regulatory T cells, Tumor microenvironment

## Abstract

The pre-metastatic niche (PMN) is a tumor-conditioned immunosuppressive microenvironment that forms in distant organs prior to metastatic colonization. Macrophages and regulatory T cells (Tregs) play central roles in its formation and function. Tumor-derived exosomes and soluble factors reprogram macrophages into M2-like states, leading to extracellular matrix (ECM) remodeling, increased angiogenesis, vascular leakiness, and fibrotic niche formation. Simultaneously, Tregs are recruited and expanded through pathways involving myeloid-derived suppressor cells (MDSCs), S100 calcium-binding protein A8/A9 (S100A8/A9), receptor for advanced glycation end products (RAGE), Toll-like receptor 4 (TLR4), as well as fibroblast-derived exosomal C-C motif chemokine ligand 1 (CCL1)-C-C chemokine receptor type 8 (CCR8) signaling. Cytokines from macrophages suppress natural killer (NK) and cluster of differentiation 8-positive (CD8^+^) T-cell cytotoxicity, facilitating immune evasion. The interaction between macrophages and Tregs creates self-reinforcing suppressive circuits that define the metastatic boundaries and dictate the fate of disseminated tumor cells toward dormancy or growth. This review summarizes the core mechanisms by which macrophages and Tregs mediate PMN formation, highlights the critical role of exosomes in macrophage reprogramming and niche boundary establishment, and discusses targeted therapeutic strategies and translational challenges.

## Highlights

• Pre-metastatic niche (PMN) biomarkers can enhance the stratification of metastasis risk in patients with cancer.
• Tumor-derived exosomes (TDEs) reprogram macrophage phenotypes to construct the PMN.
• Macrophage-regulatory T cell (Treg) crosstalk drives extracellular matrix (ECM) remodeling within the PMN.

## Graphical abstract

**Figure:**
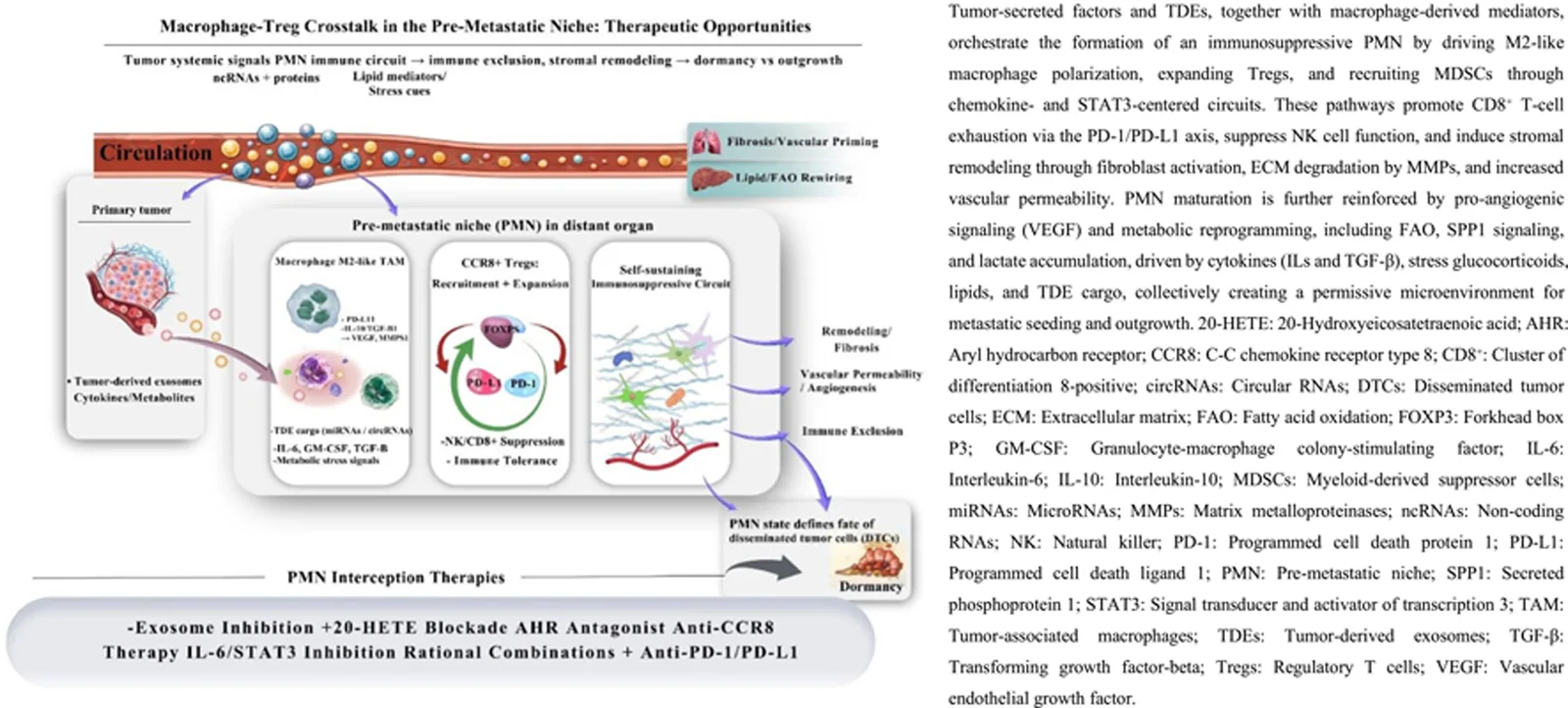

## Introduction

Metastasis remains the leading cause of cancer-related deaths, responsible for over 90% of fatalities in patients with solid tumors.^1^, ^2^, ^3^ Increasing evidence suggests that metastasis may occur as an early and coordinated process driven by tumor-host interactions and the development of the pre-metastatic niche (PMN), a tumor-conditioned microenvironment in remote organs that promotes metastatic colonization.^4^ Instead of being a passive “soil”, the PMN is actively remodeled into an immunosuppressive and pro-angiogenic microenvironment, thereby the extravasation, survival, dormancy, and growth of tumor cells. This concept expands Paget’s “seed and soil” hypothesis by highlighting the active systemic conditioning imposed by primary tumors.^5^^,^^6^ Recent research indicates that tumors can prepare future metastatic sites through systemic signals, emphasizing the need to understand PMN mechanisms.^7^

A key aspect of PMN formation involves the recruitment and reprogramming of macrophages and regulatory T cells (Tregs), which serve as primary orchestrators of early immune tolerance [Table 1].^8^, ^9^, ^10^, ^11^, ^12^ Tumor-derived exosomes (TDEs) and soluble factors induce macrophages to polarize into M2-like phenotypes, promoting extracellular matrix (ECM) remodeling, increased vascular permeability, and fibrosis through mechanisms such as lipid reprogramming, proteolysis, and pathways such as nuclear factor kappa-light-chain-enhancer of activated B cells (NF-κB)/hypoxia-inducible factor 1-alpha (HIF-1α) and Toll-like receptor 4 (TLR4). Meanwhile, Tregs expand via myeloid-derived suppressor cells (MDSCs)-S100 calcium-binding proteins A8/A9 (S100A8/A9)/receptor for advanced glycation end products (RAGE) loops, fibroblast-derived exosomal C-C motif chemokine ligand 1 (CCL1)-C-C chemokine receptor type 8 (CCR8) signaling, and macrophage cytokines, all of which work together to suppress natural killer (NK) and CD8^+^ T-cell activity and facilitate immune evasion.^4^^,^^6^^,^^13^ Consequently, PMN formation represents active immunity and stromal restructuring, rather than merely the buildup of suppressive cells.

**Table 1:** Key non-macrophage/Treg cell types involved in PMN formation.

Cell type	Role in PMN	Key mechanisms	References
Neutrophils	ECM remodeling, immunosuppression, DTC recruitment	NETosis-mediated DNA trap formation; N2 polarization via TGF-β; exosome-induced IL-8 secretion	8
MDSCs	Immune suppression, angiogenesis	S100A8/A9-mediated recruitment; suppression of NK/CD8^+^ T cells via ARG1/ROS; VEGF-driven vascularization	9, 10, 11, 12
Fibroblasts	Fibrosis, ECM deposition	Exosome-mediated activation (e.g., CCL1 secretion); fibronectin/collagen production; matrix stiffening that supports DTC adhesion	13,14
Endothelial cells	Vascular permeability, angiogenesis	Upregulation of adhesion molecules (e.g., VCAM-1); EV-induced vascular leakiness via ZO-1 degradation	15,16
Osteoclasts/Osteoblasts (Bone-specific)	Bone remodeling, DTC dormancy	RANKL/OPG imbalance; exosome-induced osteoclastogenesis; niche preparation for metastatic seeding	17,18

This table summarizes non-macrophage/Treg cellular components that contribute to PMN establishment and maturation. Mechanistic pathways frequently intersect with macrophage-Treg axes (e.g., MDSCs promote Treg expansion), underscoring the integrated nature of PMN biology. ARG1: Arginase 1; CCL1: C-C motif chemokine ligand 1; CD8^+^: Cluster of differentiation 8 positive; DTC: Disseminated tumor cell; ECM: Extracellular matrix; EVs: Extracellular vesicles; IL-8: Interleukin 8; MDSCs: Myeloid-derived suppressor cells; NETosis: Neutrophil extracellular trap formation; NK: Natural killer; OPG: Osteoprotegerin; PMN: Pre-metastatic niche; RANKL: Receptor activator of nuclear factor kappa-B ligand; ROS: Reactive oxygen species; S100A8/A9: S100 calcium-binding protein A8/A9; TGF-β: Transforming growth factor beta; Treg: Regulatory T cell; VCAM-1: Vascular cell adhesion molecule 1; VEGF: Vascular endothelial growth factor; ZO-1: Zonula occludens 1.

Mechanistically, macrophage-Treg bidirectional signaling creates self-perpetuating circuits, such as interleukin-6 (IL-6)/signal transducer and activator of transcription 3 (STAT3), granulocyte-macrophage colony-stimulating factor (GM-CSF)/signal transducer and activator of transcription 5 (STAT5)/aryl hydrocarbon receptor (AHR)/programmed death-ligand 1 (PD-L1), C-X-C motif chemokine ligand 10 (CXCL10)/C-X-C chemokine receptor type 3 (CXCR3)/TLR4, and exosomal non-coding RNAs (ncRNAs). These are reinforced by the spatial arrangement of suppressive “edge” macrophages (e.g., triggering receptor expressed on myeloid cells 2 [TREM2]^+^) and Treg clusters. These mechanisms are associated with increased metastatic burden and poorer outcomes across various tumor types.^3^^,^^7^ This review integrates insights into macrophage- and Treg-focused processes in PMN formation, highlights their crosstalk and spatial distribution, and discusses new therapeutic approaches and the challenges associated with their clinical translation.

## Pre-metastatic vs. metastatic niches

The PMN and metastatic niche form a framework where metastasis occurs through staged microenvironmental changes, rather than occurring via random hematogenous or lymphatic dissemination.^10^, ^11^, ^12^ Primary tumors prepare distant organs by releasing tumor-secreted factors and extracellular vesicles (EVs), which trigger PMN formation before tumor cells arrive.^10^^,^^13^, ^14^, ^15^, ^16^ The cargo within EVs (proteins and RNAs) enhances vascular permeability. It promotes angiogenesis, while also recruiting bone marrow-derived myeloid cells and activating fibroblasts and endothelial cells, thus creating permissive “landing sites”.^4^^,^^7^^,^^13^, ^14^, ^15^, ^16^

Importantly, EV-driven conditioning is specific to each organ: lung PMNs exhibit immune suppression and vascular priming, liver PMNs show fibrotic remodeling and ECM buildup, bone PMNs influence osteoblast and osteoclast balance, and lymph node niches promote lymphangiogenesis and immune tolerance.^10^^,^^11^^,^^16^ These results highlight the role of PMNs as an actively tumor-educated microenvironment and support prevention strategies that target EV formation, uptake, or key recruitment and signaling pathways.

In contrast, the metastatic niche develops after disseminated tumor cells (DTCs) settle and recruit local stromal and immune cells to promote survival, angiogenesis, and immune evasion.^4^^,^^17^ Tregs are frequently abundant and inhibit anti-tumor immune responses, while changes in the vasculature and stroma facilitate tumor growth.^10^^,^^11^ Overall, targeting the PMN could prevent initial seeding of disseminated tumor cells, whereas targeting the metastatic niche might restrict the growth of established lesions, suggesting a two-stage therapeutic approach.^4^^,^^16^^,^^17^

## Molecular and cellular mechanisms underlying Macrophage–Treg coordination in pre-metastatic niche (PMN) development

PMN formation relies on coordinated immune remodeling, where tumor-derived factors and exosomes steer macrophages toward M2-like states and attract or activate Tregs [Figure 1]. These cells work together to suppress NK and CD8^+^ T-cell activity, modify the ECM, and prime blood vessels, creating early immune tolerance that supports metastatic colonization.^4^^,^^7^^,^^16^^,^^17^ Below, we outline the main pathways of macrophage reprogramming and their connection to Treg recruitment and activity.

**Figure 1:**
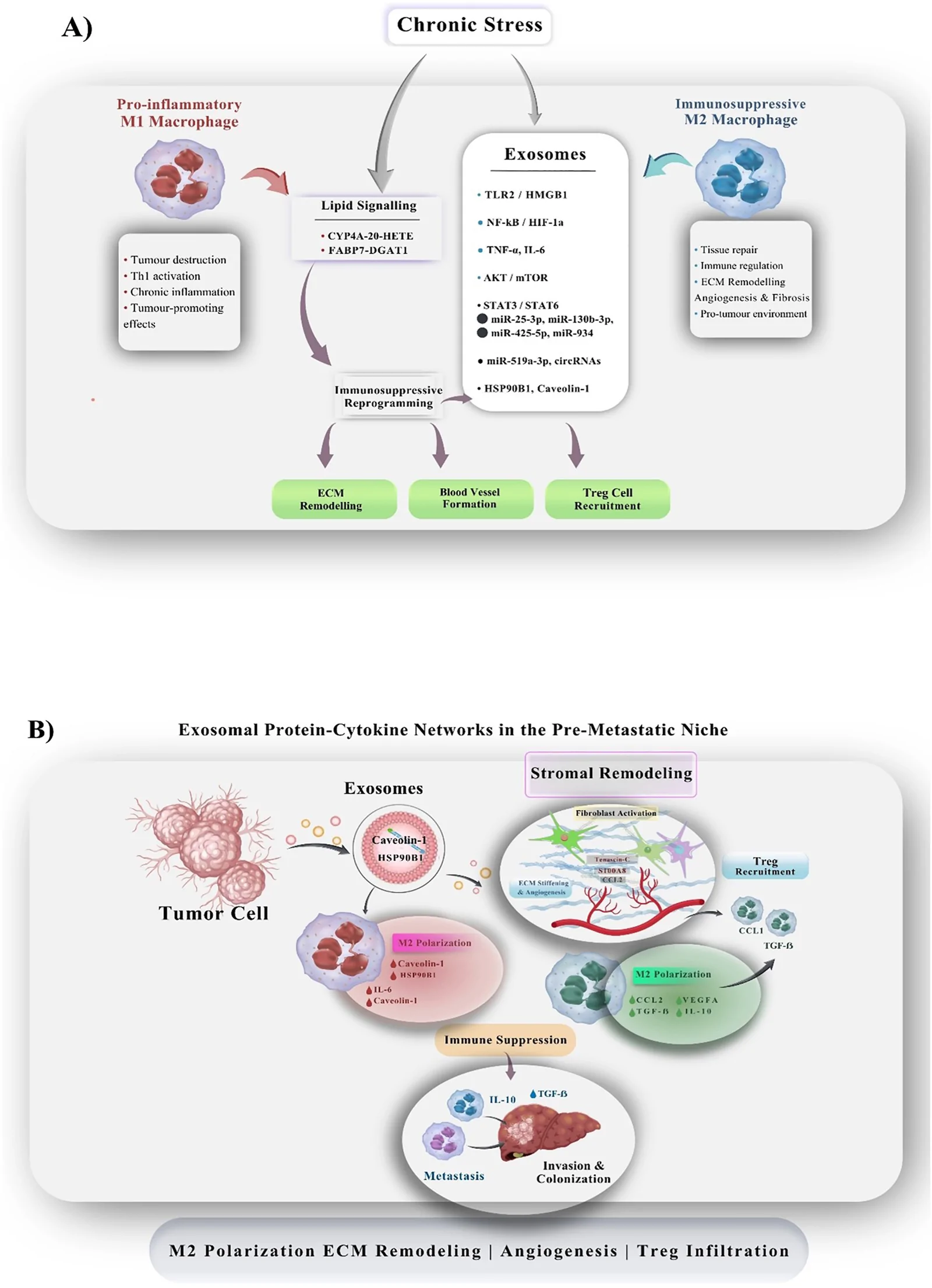
Chronic stress and exosomes mediate macrophage reprogramming in the PMN 20, 21, 22, 23^,^^39^, ^40^, ^41^, ^42^^,^^47^^,^^48^. (A) Chronic stress promotes polarization from pro-inflammatory M1 macrophages to immunosuppressive M2 macrophages through tumor-derived exosomes. M2 macrophages contribute to ECM remodeling, blood vessel formation, fibrosis, and Treg cell recruitment by releasing lipid mediators, including CYP4A-derived 20-HETE and FABP7-DGAT1-associated signaling, and by carrying molecules such as TLR2/HMGB1, NF-κB/HIF-1α, TNF-α/IL-6, AKT/mTOR, STAT3/STAT6, *miR-25-3p, miR-130b-3p, miR-425-5p, miR-519a-3p, miR-934*, circRNAs, HSP90B1, and Caveolin-1. (B) Exosomal protein-cytokine networks in the pre-metastatic niche promote M2 polarization, stromal remodeling, fibroblast activation, ECM stiffening, angiogenesis, immune suppression, invasion and colonization, and Treg recruitment. Arrows indicate the direction of signaling, cellular reprogramming, or downstream biological effects; upward arrows indicate increased expression or activity. 20-HETE: 20-hydroxyeicosatetraenoic acid; AKT: Protein kinase B; CCL1: C–C motif chemokine ligand 1; CCL2: C–C motif chemokine ligand 2; circRNAs: Circular RNAs; COL12: Collagen type XII; CYP4A: Cytochrome P450 family 4 subfamily A; DGAT1: Diacylglycerol O-acyltransferase 1; ECM: Extracellular matrix; FABP7: Fatty acid-binding protein 7; HIF-1α: Hypoxia-inducible factor 1-alpha; HMGB1: High mobility group box 1; HSP90B1: Heat shock protein 90 beta family member 1; IL-6: Interleukin 6; IL-10: Interleukin 10; M1: Classically activated macrophage; M2: Alternatively activated macrophage; *miR-25-3p*: MicroRNA-25-3p; *miR-130b-3p*: MicroRNA-130b-3p; *miR-425-5p*: MicroRNA-425-5p; *miR-519a-3p*: MicroRNA-519a-3p; *miR-934*: MicroRNA-934; mTOR: Mammalian target of rapamycin; NF-κB: Nuclear factor kappa-light-chain-enhancer of activated B cells; PMN: Pre-metastatic niche; S100A8: S100 calcium-binding protein A8; STAT3: Signal transducer and activator of transcription 3; STAT6: Signal transducer and activator of transcription 6; ST6Gal1: ST6 beta-galactoside alpha-2,6-sialyltransferase 1; TGF-β: Transforming growth factor beta; Th1: T helper 1; TLR2: Toll-like receptor 2; TNF-α: Tumor necrosis factor alpha; Treg: Regulatory T cell; VEGFA: Vascular endothelial growth factor A.

### Lipid-derived pathways governing macrophage polarization

Lipid signaling is a key factor in macrophage reprogramming within PMNs. The cytochrome P450 4A (CYP4A)-20-hydroxyeicosatetraenoic acid (20-HETE) pathway enhances STAT3 activation in tumor-associated macrophages (TAMs). It stimulates the production of transforming growth factor-β (TGF-β), vascular endothelial growth factor (VEGF), and stromal cell-derived factor 1 (SDF-1), which promote the recruitment of VEGF receptor 1-positive (VEGFR1^+^) myeloid cells, activate fibroblasts, induce fibronectin buildup, and increase vascular permeability, thereby supporting extravasation.^18^ Blocking CYP4A or STAT3 decreases these PMN processes and reduces metastasis in preclinical models.^18^, ^19^, ^20^

Beyond 20-HETE, fatty acid oxidation (FAO) and peroxisome proliferator-activated receptor gamma (PPARγ)-related programs support M2-like phenotypes. Tumor exosomes and cancer-associated fibroblast (CAF) signals promote lipid accumulation and suppress T-cell activity through mediators like fatty acid-binding proteins 5 and 7 (FABP5/7) and phosphoinositide 3-kinase gamma (PI3Kγ).^21^, ^22^, ^23^, ^24^, ^25^ Exosomal microRNAs (miRNAs) and macrophage regulators (e.g., S100 calcium-binding protein A4 [S100A4] and V-set and immunoglobulin domain-containing 4 [VSIG4]) strengthen FAO-dependent suppression.^21^^,^^23^ Targeting lipid metabolic pathways can reprogram macrophages and decrease metastatic potential.^22^^,^^24^^,^^25^

### Lipid droplets (LDs) and immunometabolic conditioning

Lipid droplet buildup in macrophages is increasingly associated with PMN immunosuppression and metastasis, especially in liver-tropic environments.^22^, ^23^, ^24^, ^25^, ^26^, ^27^, ^28^, ^29^ Hypoxia-induced fatty acid-binding protein 7 (FABP7) boosts diacylglycerol O-acyltransferase 1 (DGAT1) activity, leading to lipid droplet creation and FAO, which supports macrophage polarization toward the M2-like state. The transfer of lipids to CD8^+^ T cells results in exhaustion, indicated by increased programmed cell death protein 1 (PD-1), T-cell immunoglobulin and mucin domain-containing protein 3 (TIM-3), and decreased production of interferon-gamma (IFN-γ), tumor necrosis factor-alpha (TNF-α), and granzyme B, thereby facilitating tumor metabolic adaptation.^22^^,^^23^ Macrophages rich in lipid droplets (LDs) also attract Tregs through chemokines like C–C motif chemokine ligand 20 (CCL20)-C-C chemokine receptor type 6 (CCR6) and maintain their suppressive functions via FAO/mechanistic target of rapamycin (mTOR) pathways.^24^, ^25^, ^26^, ^27^, ^28^ Blocking lipid droplet formation or its regulators (including DGAT1, FABP4/7, and PI3Kγ) diminishes Treg infiltration and tumor growth in experimental models.^22^^,^^24^^,^^26^, ^27^, ^28^, ^29^

### Extracellular matrix (ECM) remodeling by macrophages

Macrophages remodel the ECM by secreting matrix metalloproteinases (MMPs) (e.g., MMP-2/9/14) and through mechanical invasion, which increases vascular permeability and creates migration tracks that tumor cells can use.^30^^,^^31^ In the PMN, macrophage recruitment activates fibroblasts and promotes ECM deposition, crosslinking, and stiffening (e.g., via S100A4/extracellular signal-regulated kinase [ERK]), supporting colonization and immune evasion.^30^, ^31^, ^32^, ^33^ Macrophages also help recruit Tregs by upregulating PD-L1, often through AHR signaling, while Tregs promote M2-like polarization by suppressing inflammatory signals, thus maintaining fibrosis and immune tolerance.^10^^,^^13^^,^^30^, ^31^, ^32^

### Neuroendocrine stress pathways

Chronic stress enhances PMN conditioning by strengthening TAM-MDSC suppressive networks. Activation of glucocorticoid receptors in TAMs upregulates C-X-C motif chemokine ligand 1 (CXCL1), which recruits splenic MDSCs to pre-metastatic lungs through C-X-C chemokine receptor type 2 (CXCR2), thereby suppressing cytotoxic immunity and aiding metastatic colonization.^34^, ^35^, ^36^, ^37^, ^38^ Additionally, β-adrenergic signaling promotes MDSC mobilization and boosts suppression via the C-X-C motif chemokine ligand 5 (CXCL5)-CXCR2-ERK and IL-6/STAT3 pathways, while also increasing FAO and prostaglandin E2 (PGE2) production.^35^, ^36^, ^37^, ^38^ Interventions that inhibit β-adrenergic signaling, TAMs, or the CXCL1-CXCR2 axis have been shown to decrease PMN formation and metastasis in experimental models.^34^, ^35^, ^36^, ^37^

### Exosome-induced metabolic rewiring

TDEs can reprogram macrophages to favor glycolysis through TLR2/high-mobility group box 1 (HMGB1)–NF-κB and HIF-1α/glucose transporter 1 (GLUT1) pathways. This increases lactate production, PD-L1 expression, and suppressive functions in cluster of differentiation 206-positive (CD206^+^) macrophages, leading to T-cell exhaustion, recruitment of MDSCs, acidification of the ECM, and vascular leakiness.^39^^,^^40^ Similar exosome-mediated signaling in various cancers influences pathways such as NF-κB, PI3K/protein kinase B (AKT)/mTOR, STAT3/STAT6, and Hippo/Yes-associated protein (YAP), which support macrophage adaptability and early niche development.^41^^,^^42^ Exosomes also condition macrophages to produce IL-10 and TGF-β, which facilitate Treg recruitment and maintain suppressive feedback loops.^39^, ^40^, ^41^, ^42^

### Exosomal non-coding RNAs (ncRNAs)

Exosomal miRNAs and circular RNAs (circRNAs) promote macrophage M2 polarization by inhibiting regulators like phosphatase and tensin homolog (PTEN) and dual-specificity phosphatase 2 (DUSP2) while activating PI3K/AKT and mitogen-activated protein kinase (MAPK)/ERK signaling pathways. This leads to increased production of pro-angiogenic and immunosuppressive mediators such as TGF-β and VEGFs, as well as enhanced vascular permeability and ECM remodeling. Exosomal circRNAs, including *circATP8A1* and *circ_0034880*, function as miRNA sponges that activate the STAT6/Janus kinase (JAK)-STAT pathway and related signaling, promoting secreted phosphoprotein 1 (*SPP1*)-high CD206^+^ macrophage phenotypes and liver PMN formation. Additionally, macrophage-derived exosomes can induce Treg differentiation—for example, through *miR-4443*-mediated mechanisms—further strengthening immune suppression.^43^, ^44^, ^45^, ^46^

### Exosomal protein-cytokine networks

Exosomal proteins such as heat shock protein 90 beta family member 1 (HSP90B1) and Caveolin-1 facilitate M2-like polarization, stromal activation, Tenascin-C-dependent ECM buildup, and increased vascular permeability, often collaborating with IL-6/STAT3 signaling.^47^^,^^48^ Transcription factor (ETS1)-controlled exosomal laminins can induce reprogramming of omental macrophages and promote chemokine release that enhances organ-specific tropism.^49^ These mechanisms also attract Tregs, especially through fibroblast CCL1-CCR8 signaling, and strengthen IL-10/TGF-β loops that support immune evasion [Figure 1].^13^^,^^46^, ^47^, ^48^, ^49^

### TRAIL-driven reprogramming under glucose restriction

Under glucose restriction, endoplasmic reticulum (ER) stress induces HMG-CoA reductase degradation protein 1 (HRD1)-dependent lysine 63 (K63) ubiquitination of tumor necrosis factor-related apoptosis-inducing ligand (TRAIL) and endosomal sorting complexes required for transport (ESCRT)-mediated loading into exosomes, facilitating their systemic spread to distant organs.^50^ In lung PMNs, exosomal TRAIL reprograms poliovirus receptor (PVR; CD155)^+^ macrophages toward an immunosuppressive state, marked by increased PD-L1 and decreased IFN-γ, which contributes to NK cell dysfunction and promotes metastasis. In experimental models, neutralizing TRAIL restores NK cell activity and reduces metastasis, while combining this approach with T-cell immunoreceptor with immunoglobulin and immunoreceptor tyrosine-based inhibitory motif domain (TIGIT) blockade further improves outcomes. This reveals a targetable pathway in glucose-stressed tumors like lung adenocarcinoma.^50^ These results connect tumor metabolic stress to immune alterations in distant sites via exosome signaling and complement the metabolic and exosomal mechanisms detailed earlier.

### Integration of macrophage reprogramming mechanisms

Macrophage reprogramming pathways converge on common hubs, including HIF-1α, NF-κB, and STAT3, that combine EV signals, metabolic stress, and inflammatory cues into self-reinforcing suppressive networks.^51^^,^^52^ EV cargo can enhance FAO and checkpoint regulation, maintaining STAT3 activity and PD-L1 expression while promoting Treg accumulation. For instance, exosomal *miR-4488* supports FAO-linked suppression through reticulon 3 (RTN3)/FABP5-related pathways, reinforcing PD-L1-mediated immune exclusion and macrophage-Treg feedback.^23^ Systemic factors like neuroendocrine stress may further amplify these circuits by enhancing inflammatory transcriptional programs and EV secretion, thereby integrating metabolic, vesicular, and inflammatory pathways into a coordinated PMN-conditioning network.^34^, ^35^, ^36^, ^37^, ^38^, ^39^, ^40^, ^41^, ^42^^,^^51^^,^^52^ This convergence explains the limited effectiveness of single-target interventions and underscores the need for multi-target strategies that disrupt these central hubs and break macrophage-Treg reinforcement loops.^51^^,^^52^

### Organ-specific Macrophage–Treg regulatory mechanisms

Macrophage–Treg interactions differ across organs because of variations in immune cell populations, stromal structure, blood vessels, and metabolic factors. In lung PMNs, chemokines like CCL1-CCR8 frequently drive vascular priming and immune exclusion, leading to CCR8^+^ Treg accumulation and macrophage checkpoint activation in hypoxic or inflamed areas.^4^^,^^13^ In the liver, lipid-induced FAO reprogramming, such as FABP7-related pathways, within Kupffer and monocyte-derived macrophages creates chemokine gradients (e.g., CCL20-CCR6) that attract Tregs and facilitate fibrotic remodeling.^22^, ^23^, ^24^, ^25^ In bone tissue, macrophage-osteoclast pathways interact with receptor activator of nuclear factor kappa-B ligand (RANKL)-driven turnover and Treg suppression, aiding dissemination and the transition from dormancy to growth.^16^^,^^17^ In brain environments, microglia-dominated immunoregulatory states and Treg-produced mediators (e.g., IL-10) reduce cytotoxic immune responses under hypoxic or metabolically restricted conditions.^4^^,^^6^ These regional variations suggest that combining shared pathway blockade (such as STAT3/NF-κB/AHR inhibitors) with organ-specific treatments targeting the dominant local mechanisms can be effective.

## Key signaling pathways driving Treg-macrophage crosstalk

Building on the preceding discussion of molecular and cellular mechanisms, we summarize the key signaling pathways that facilitate macrophage-Treg crosstalk and reinforce PMN immune suppression [Figure 2]. These pathways integrate cytokine signaling, metabolic reprogramming, and stromal feedback loops, thereby promoting reciprocal activation between macrophages and Tregs. Table 2 offers a concise overview and distinguishes between evidence that is causal (derived from perturbation studies) and associative (obtained from correlative profiling).

**Figure 2:**
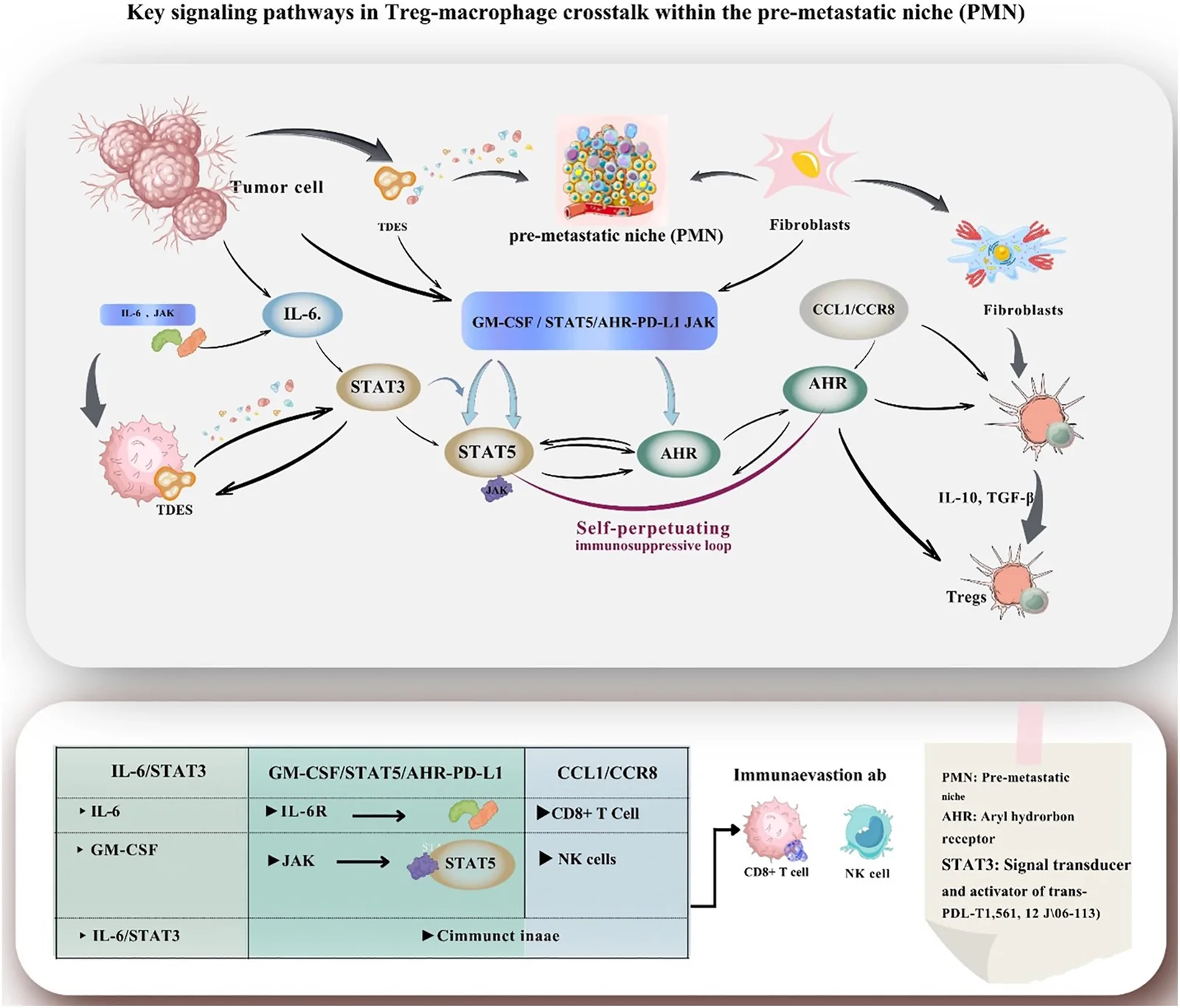
Therapeutic targeting of macrophage–Treg interactions in the PMN ^10^^,^^13^^,^^39^, ^40^, ^41^, ^42^, ^43^, ^44^, ^45^, ^46^, ^47^, ^48^, ^49^^,^^52^, ^53^, ^54^^,^^56^, ^57^, ^58^, ^59^, ^60^, ^61^, ^62^. Tumor-derived systemic signals, including TDEs, cytokines, metabolites, ncRNAs, lipid mediators, and stress-related factors, condition distant tissues and promote the formation of an immunosuppressive PMN. Within this microenvironment, macrophage- and fibroblast-associated signaling pathways, including IL-6/STAT3, GM-CSF/STAT5/AHR/PD-L1, and CCL1/CCR8, support Treg recruitment and amplification, suppress CD8^+^ T-cell and NK-cell effector functions, and promote immune evasion. M2-like tumor-associated macrophages and CCR8^+^ Tregs further enhance immunosuppression through mediators such as IL-10 and TGF-β1, thereby facilitating stromal remodeling and metastatic colonization. The lower panel summarizes representative therapeutic strategies targeting the IL-6/STAT3, GM-CSF/STAT5/AHR/PD-L1, and CCL1/CCR8 axes. Arrows indicate the direction of signaling, recruitment, or downstream biological effects, and the purple curved arrow indicates a self-perpetuating immunosuppressive feedback loop. 20-HETE: 20-hydroxyeicosatetraenoic acid; AHR: Aryl hydrocarbon receptor; CCL1: C–C motif chemokine ligand 1; CCR8: C–C chemokine receptor type 8; CD8^+^: Cluster of differentiation 8-positive; EVs: Extracellular vesicles; GM-CSF: Granulocyte-macrophage colony-stimulating factor; IL-6: Interleukin 6; IL-10: Interleukin 10; JAK: Janus kinase; MMPs: Matrix metalloproteinases; ncRNAs: Non-coding RNAs; NK: Natural killer cells; PD-1: Programmed cell death protein 1; PD-L1: Programmed death-ligand 1; PMN: Pre-metastatic niche; STAT3: Signal transducer and activator of transcription 3; STAT5: Signal transducer and activator of transcription 5; TAMs: Tumor-associated macrophages; TDEs: Tumor-derived exosomes; TGF-β1: Transforming growth factor beta 1; Tregs: Regulatory T cells; VEGF: Vascular endothelial growth factor.

**Table 2:** Signaling pathways in macrophage-Treg crosstalk.

Pathway	Key components	Role in PMN	Causality evaluation	Ref
IL-6/STAT3	IL-6 activates JAK/STAT3 signaling and promotes PD-L1 upregulation	Attracts MDSCs, skews M2, suppresses T/NK cells	Causal: STAT3 KO reverses effects *in vivo*; associative in human correlations	52, 53, 54
CCL1-CCR8	Exosomal CCL1 released from fibroblasts binds to CCR8 on T cells	Treg differentiation/recruitment	Causal: CCR8 blockade reduces Tregs/metastasis; associative in expression data	13,55, 56, 57, 58
GM-CSF–STAT5–AHR–PD-L1	GM-CSF activates STAT5/AHR signaling and induces PD-L1 expression in macrophages	Treg induction, T-cell exhaustion	Causal: AHR deletion inhibits Treg expansion; associative in prognostic links	10,59, 60, 61, 62
Spatial coordination	TREM2^*+*^ macrophages and Treg clusters localize at invasive edges	Immune exclusion	Associative: Spatial profiling correlations; causal via depletion studies	67, 68, 69

Causality was assessed via functional studies (e.g., KO, inhibitors) vs. associations (e.g., correlations). AHR: Aryl hydrocarbon receptor; CCL1: C–C motif chemokine ligand 1; CCR8: C–C chemokine receptor type 8; GM-CSF: Granulocyte-macrophage colony-stimulating factor; IL-6: Interleukin 6; JAK: Janus-activated kinase; KO: Knockout; M2: M2-like macrophage; MDSCs: Myeloid-derived suppressor cells; NK: Natural killer; PD-L1: Programmed death-ligand 1; PMN: Pre-metastatic niche; STAT3: Signal transducer and activator of transcription 3; STAT5: Signal transducer and activator of transcription 5; Treg: Regulatory T cell; TREM2: Triggering receptor expressed on myeloid cells 2.

### Interleukin 6 (IL-6)/Signal transducer and activator of transcription 3 (STAT3)

IL-6/STAT3 signaling enhances immunosuppressive conditions in lung PMNs by activating STAT3 within tumor cells, leading to increased epithelial-mesenchymal transition (EMT)-like programs and PD-L1 expression, and by altering immune cell composition through increased granulocytic MDSCs, M2-like macrophage skewing, and decreased CD8^+^/NK activity.^52^, ^53^, ^54^ In non-small cell lung cancer (NSCLC), higher IL-6/STAT3 activity is linked to increased PD-L1 levels, more MDSCs and Tregs, fewer CD8^+^ T cells and worse patient outcomes; tumor-intrinsic PD-L1 can further activate IL-6/JAK/STAT3 pathways, contributing to resistance against checkpoint inhibitors.^52^, ^53^, ^54^ While perturbation studies indicate causality *in vivo*, human data are mostly correlational.

### C-C motif chemokine ligand 1 (CCL1)-C-C chemokine receptor type 8 (CCR8)

Tumor exosomes reprogram lung fibroblasts to boost CCL1 secretion, which binds to CCR8 on T cells and enhances forkhead box P3-positive (Foxp3^*+*^) Treg differentiation prior to tumor cell arrival.^13^^,^^55^, ^56^, ^57^, ^58^ CCR8^+^ Tregs are highly suppressive and linked to poor prognosis; blocking CCR8 lowers Treg infiltration and metastasis in models.^56^, ^57^, ^58^

### Granulocyte-macrophage colony-stimulating factor-signal transducer and activator of transcription 5-aryl hydrocarbon receptor-programmed death-ligand 1 (GM-CSF–STAT5–AHR–PD-L1)

Tumor GM-CSF activates STAT5 and stabilizes AHR in macrophages, leading to increased PD-L1 expression and Treg recruitment.^10^^,^^59^, ^60^, ^61^, ^62^ Additional signals (CXCL10-CXCR3; TLR4) recruit MDSCs and promote Treg expansion, while Tregs support M2-like programs through IL-10/TGF-β/adenosine. These interactions create a PD-L1/PD-1 feedback loop that suppresses cytotoxic T/NK cell activity and facilitates metastasis, with strong causal evidence in preclinical models but mainly associative human prognostic data.^10^^,^^59^, ^60^, ^61^, ^62^

### Tumor-associated regulatory T cells (TA-Tregs) in dynamic microenvironment remodeling and macrophage interactions

Tumor-associated Tregs (TA-Tregs) can expand early at future metastatic sites, acting as “organizers” of suppressive immune environments. In syngeneic lung metastasis models, single-cell and spatial analyses reveal that TA-Tregs with strong suppressive and trafficking signatures—such as Foxp3-related programs with CCR8/CCL5-enriched phenotypes—appear in the lung premetastatic niche before tumor cells are visible and interact spatially with myeloid populations.^63^, ^64^, ^65^, ^66^ These early contacts between Tregs and myeloid cells align with macrophage transitions toward inflammatory or monocyte-like states, such as *Vcan*-associated states. This suggests that Tregs actively influence macrophage phenotypes during niche maturation rather than merely accumulating after myeloid remodeling. Trajectory analyses further support a gradual co-evolution of tumor-associated macrophage (TAM) and Treg states throughout metastatic progression.^65^^,^^66^ In human lung adenocarcinoma, the spatial co-localization of suppressive Tregs with TAM-rich areas, along with reduced effector-cell activity, indicates a conserved pro-metastatic regulatory mechanism. However, much of the human evidence remains correlational.^67^, ^68^, ^69^

### Tumor-derived exosomes (TDEs) orchestrate Macrophage–Treg crosstalk

TDEs are key upstream regulators of macrophage-Treg circuits in the PMN by linking metabolic changes to checkpoint reinforcement. When macrophages take up TDEs, they activate TLR2/myeloid differentiation primary response 88 (MyD88)–NF-κB signaling and induce a glycolytic shift through HIF-1α/GLUT1-dependent glucose uptake, increased lactate production, and sustained NF-κB activity. This leads to significant PD-L1 upregulation on macrophages.^39^^,^^40^ The increased PD-L1 promotes Treg differentiation and expansion, establishing early immune tolerance in metastatic target tissues and creating a regulatory-dominant microenvironment.^10^^,^^39^^,^^40^ Simultaneously, exosomal ncRNAs and proteins support M2-like polarization, boost IL-10 and TGF-β production, and enhance chemokine-driven Treg recruitment. These processes form feed-forward loops that stabilize immune escape and promote metastatic growth.^43^, ^44^, ^45^, ^46^, ^47^, ^48^, ^49^ Overall, TDEs-driven programming of macrophages acts as a mechanistic link between systemic tumor signals and the local development of suppressive macrophage-Treg circuits in the PMN.

### Spatial coordination in the pre-metastatic niche (PMN)

The spatial arrangement of immune cells plays a key role in immune exclusion and the promotion of metastasis. In lung cancer, multiplex imaging and spatial profiling reveal that suppressive macrophage subsets, including TREM2^+^ populations, tend to cluster at invasive edges and stromal-tumor boundaries. There, they act as regulatory barriers that limit CD8^+^ T-cell access and activity. Tregs often co-localize with these macrophage-rich areas, and a close spatial relationship among macrophages, Tregs, and CD8^+^ T cells is linked to poorer clinical outcomes and lower responses to immunotherapy. These findings suggest that the spatial distribution of suppressive immune cells is as critical as their abundance for prognostic prediction. This supports the use of spatially informed biomarkers and interventions that focus on breaking down suppressive neighborhoods, rather than solely depleting specific cell types. 67, 68, 69

### Integrated Macrophage–Treg circuits: a conceptual framework

Across models, macrophages and Tregs operate as interconnected regulatory modules that form closed feedback loops, thereby enhancing immune suppression [Figure 3]. A typical circuit involves macrophage checkpoint induction (notably PD-L1), which promotes Treg expansion and/or stabilization, while Treg-derived mediators (such as IL-10 and TGF-β) further reinforce M2-like macrophage programming and suppress cytotoxic effector function.^64^, ^65^, ^66^ Perturbation studies support causality for key nodes of this circuit *in vivo* (for example, checkpoint blockade or pathway inhibition decreases suppressive macrophage activity and Treg accumulation), whereas many human datasets mainly show associations through co-localization and outcome correlations.^10^^,^^56^, ^57^, ^58^, ^59^, ^60^, ^61^, ^62^, ^63^, ^64^, ^65^, ^66^, ^67^, ^68^, ^69^ This circuit model predicts that targeting a single node can be bypassed due to redundancy, and it supports rational combination approaches that disrupt convergent control points (such as concurrent blockade of macrophage checkpoint programming and Treg trafficking or survival pathways).^64^, ^65^, ^66^, ^67^, ^68^, ^69^

**Figure 3:**
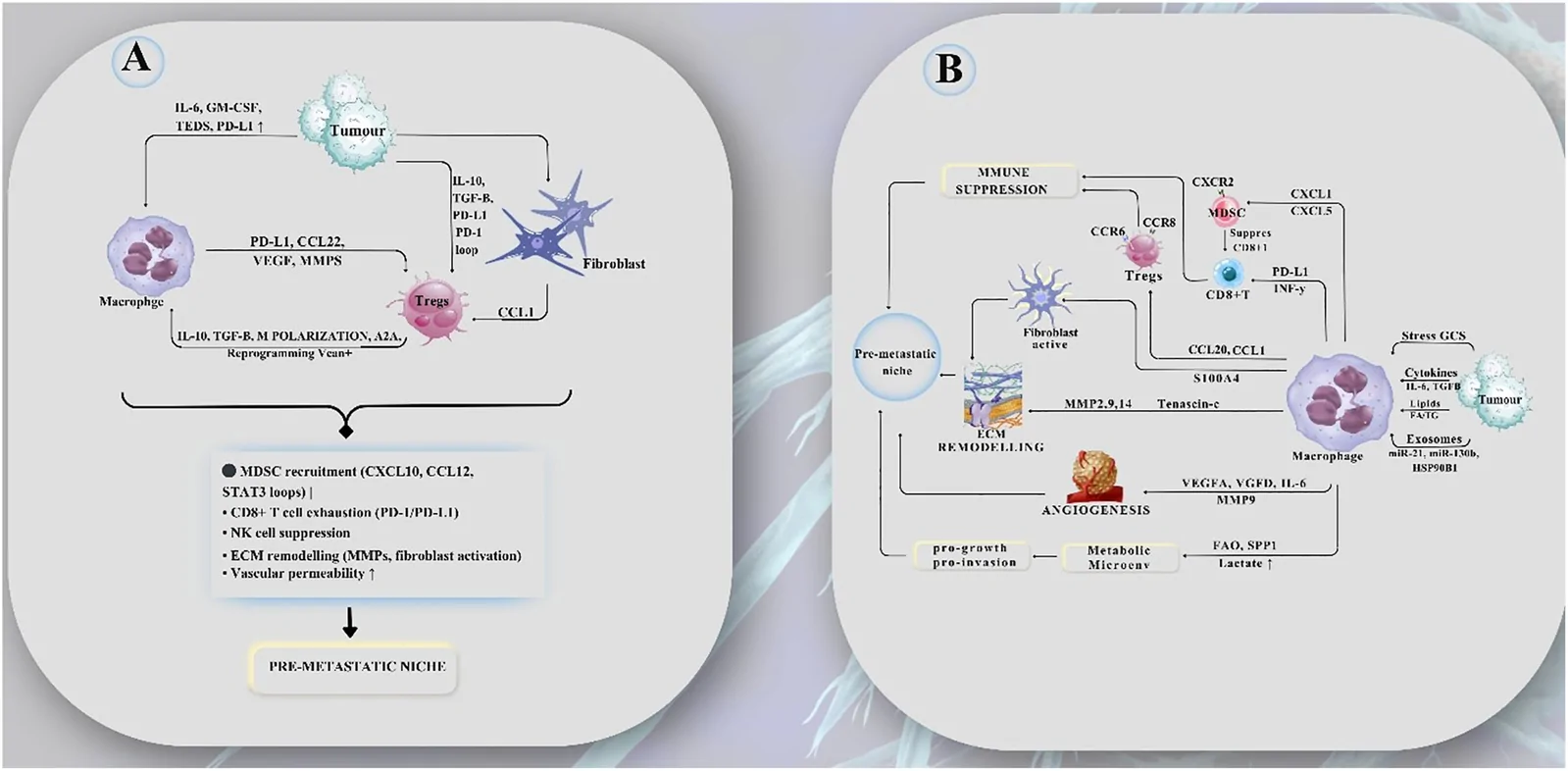
Signaling pathways and feedback loops governing macrophage–Treg communication within the PMN ^10^^,^^13^^,^^39^, ^40^, ^41^, ^42^, ^43^, ^44^, ^45^, ^46^, ^47^, ^48^, ^49^, ^50^^,^^52^, ^53^, ^54^^,^^64^, ^65^, ^66^, ^67^, ^68^, ^69^. TDEs deliver IL-6, GM-CSF (A), and other cargo to activate IL-6-JAK/STAT3, GM-CSF–STAT5–AHR–PD-L1, and CCL1/CCR8 pathways (B). These pathways strengthen macrophage-Treg feedback (with increased IL-10, TGF-β), suppress NK/CD8^+^ effector functions, activate fibroblasts, and stabilize PMN immune suppression to support metastatic seeding. AZA: 5-azacytidine; CCL1: C–C motif chemokine ligand 1; CCL12: C–C motif chemokine ligand 12; CCL20: C–C motif chemokine ligand 20; CCL22: C–C motif chemokine ligand 22; CCR2: C–C chemokine receptor type 2; CCR6: C–C chemokine receptor type 6; CCR7: C–C chemokine receptor type 7; CCR8: C–C chemokine receptor type 8; CD8^+^: Cluster of differentiation 8 positive; CXCL1: C-X-C motif chemokine ligand 1; CXCL10: C-X-C motif chemokine ligand 10; CXCL5: C-X-C motif chemokine ligand 5; CXCR2: C-X-C chemokine receptor type 2; ECM: Extracellular matrix; FAD: Flavin adenine dinucleotide; GCS: Glucocorticoid; GM-CSF: Granulocyte-macrophage colony-stimulating factor; IL-10: Interleukin 10; IL-6: Interleukin 6; IFN-γ: Interferon-gamma; MDSC: Myeloid-derived suppressor cell; MMP2: Matrix metalloproteinase 2; MMP9: Matrix metalloproteinase 9; MMP14: Matrix metalloproteinase 14; MMPs: Matrix metalloproteinases; NK: Natural killer cells; PD-L1: Programmed death-ligand 1; S100A4: S100 calcium-binding protein A4; SPP1: Secreted phosphoprotein 1 (osteopontin); STAT3: Signal transducer and activator of transcription 3; TDEs: Tumor-derived exosomes; TGF-β: Transforming growth factor beta; Tregs: Regulatory T cells; VEGF: Vascular endothelial growth factor; VEGFA: Vascular endothelial growth factor A; VEGFD: Vascular endothelial growth factor D.

### Current disputes in pre-metastatic niche (PMN) biology

The key unresolved issue is how Tregs are maintained in PMNs—through local proliferation of resident Tregs, systemic recruitment, or both. Evidence favoring recruitment comes from mechanistic studies showing that tumor-derived factors such as exosomes can reprogram stromal cells (e.g., fibroblasts) to generate chemokine gradients such as CCL1, which promote trafficking and differentiation of CCR8^+^ suppressive Tregs in target tissues.^13^^,^^55^, ^56^, ^57^, ^58^ Conversely, longitudinal immune remodeling studies during metastatic progression support scenarios involving progressive enrichment and *in situ* expansion of suppressive Treg states during niche maturation, although direct lineage evidence remains limited in many systems.^65^^,^^66^ These mechanisms likely coexist and may vary depending on tumor type, organ environment, inflammatory conditions, and experimental models. Furthermore, different profiling methods (such as flow cytometry, single-cell RNA sequencing [scRNA-seq], and spatial mapping) capture distinct aspects of Treg cycling, localization, and function.^7^^,^^63^, ^64^, ^65^, ^66^, ^67^, ^68^, ^69^ Resolving this debate will require integrated experimental designs that directly measure recruitment vs proliferation, such as fate mapping or lineage tracing combined with longitudinal sampling and spatial multi-omics. These studies would also help inform therapeutic strategies—such as blocking the chemokine axis in recruitment-dominant niches vs targeting local Treg fitness or survival in proliferation-dominant niches—and clarify whether Treg maintenance follows shared principles or context-specific rules.

## Cross-cutting themes: Treg interactions beyond macrophages

Tregs suppress effector immunity and coordinate immune networks across metastatic phases.^64^, ^65^, ^66^ Here, we emphasize stage-dependent functional effects (with mechanistic overlap discussed earlier in the parts on macrophage reprogramming and macrophage–Treg signaling pathways).

### Natural killer (NK) cells

In lung PMNs, higher Treg levels are linked with lower NK cell abundance and activity (e.g., decreased cluster of differentiation 107 [CD107]), indicating early immune suppression.^65^^,^^66^ Similar trends are observed during disease progression in metastatic settings, reinforcing a consistent Treg-NK suppressive relationship.^65^^,^^66^

### Conventional T cells

In tumor-draining lymph nodes (TDLNs), Tregs exhibit enhanced Foxp3-related programs, whereas CD4^+^/CD8^+^ T cells display diminished inflammatory and interferon responses, indicating early tolerance. In metastatic tissues, proliferating Tregs expand together with differentiated CD8^+^ T cells, yet overall effector functions remain suppressed; IL-6/STAT3 pathways also contribute to immune exclusion and decreased expression of cytotoxic markers.^53^^,^^54^^,^^65^^,^^66^

### B cells

In TDLNs, Treg enrichment is associated with a decrease in marginal zone B cells and a suppression of the PI3K-AKT-mTOR and TNFα–NF-κB pathways, indicating impaired B-cell maturation and proliferation during early remodeling.^70^ More broadly, Foxp3^+^ Tregs can promote CD8^+^ T-cell dysfunction within tumor microenvironments and may influence responses to immune checkpoint inhibitors, linking Treg enrichment to impaired anti-tumor effector immunity.^71^ In metastatic lungs, regulatory B cells (Bregs) expand across different stages and might help maintain environments that favor tumor growth.^65^^,^^66^

### Organ-specific regulatory mechanisms in pre-metastatic niche (PMN) formation

Macrophage–Treg programs are context-dependent and vary across organs because of differences in local immune composition, stromal architecture, vascular biology, and metabolic constraints. In the lung, PMN priming typically involves early vascular leakiness and immune exclusion, supported by fibroblast-immune signaling such as the CCL1-CCR8 axis that enriches CCR8^*+*^ Tregs and strengthens macrophage checkpoint programs under hypoxic and inflammatory conditions.^4^^,^^13^^,^^55^, ^56^, ^57^, ^58^ In the liver, PMN conditioning is closely linked to immunometabolism: lipid droplet buildup and FAO reprogramming in macrophage subsets (including FABP7-associated programs) can create chemokine gradients (e.g., CCL20-dependent signaling) that promote Treg recruitment and fibrotic remodeling.^22^, ^23^, ^24^, ^25^ In bone, immune suppression overlaps with skeletal turnover; RANKL/osteoprotegerin (OPG)-regulated osteoclast activity and macrophage-mediated stromal remodeling work with Treg-associated suppression to support DTCs’ persistence and transitions from dormancy to growth.^16^^,^^17^

In the brain, microglia-driven immune surveillance and limited immune access foster immunoregulatory states; Treg-derived mediators (e.g., IL-10) can further suppress cytotoxic programs in metabolically constrained, hypoxic environments.^4^^,^^6^ Overall, these organ-specific differences suggest a dual approach for PMN management (i) targeting shared pathways (e.g., STAT3/NF-κB/AHR); and (ii) implementing organ-specific interventions aimed at the main local drivers of immune suppression and stromal remodeling.^4^^,^^6^^,^^7^

## Therapeutic implications and challenges

PMN interception aims to disrupt macrophage-Treg regulatory circuits that drive immune suppression, vascular priming, and extracellular matrix remodeling in distant organs. Table 3 summarizes key targets and candidate biomarkers. Major challenges include timing, organ-specific differences, and pathway redundancy, supporting rational combinations instead of single-agent approaches.

**Table 3:** Core therapeutic targets and candidate biomarkers for disrupting macrophage-Treg programs and PMN-relevant detection/interception.

Type	Name/biomarker	Mechanistic link/underlying mechanism	Approach/sample type	Phase/performance	Outcomes/value & limitations	Ref/Trial ID
Target	CCR8	Depletes CCR8^*+*^ suppressive Tregs enriched in tumors; reduces immune exclusion	Fc-optimized anti-CCR8 mAbs (e.g., BAY 3375968, CHS-114)	Phase I/II	Early data: evidence of intratumoral Treg reduction; ongoing safety/efficacy	56, 57, 58; NCT05537740; NCT05635643
Target	CSF1R	Inhibits CSF1R signaling and reduces M2-like macrophage programs and myeloid recruitment	CSF1R inhibitors/antibodies (e.g., axatilimab/SNDX-6352, pexidartinib)	Phase I-III (various combination regimens)	Feasibility in combinations; hepatotoxicity/inflammation reported	83,84; NCT04301778
Target	CD47 (magrolimab)	Blocks the “don’t eat me” signal and enhances phagocytosis by macrophages	Anti-CD47 mAb + chemotherapy/ICIs	Phase I-III (solid tumors; some programs halted hematologic malignancies)	Activity in some solid tumors; anemia risk	86,87; NCT02953782
Target	AHR	GM-CSF/STAT5 signaling stabilizes AHR, which promotes PD-L1 expression in macrophages and induces Tregs	Small-molecule AHR inhibitors (preclinical leads)	Preclinical	Reduces Treg expansion/metastasis in models; toxicity concerns	10,59,60
Target	CYP4A/20-HETE	Lipid signaling activates STAT3 and promotes M2-like polarization, ECM remodeling, and vascular changes	CYP4A inhibitors (e.g., HET0016; preclinical)	Preclinical	Reduced metastasis in models; toxicity/translation unknown	18, 19, 20,88
Target	Exosomal cargo (e.g., HSP90B1, circRNAs)	EV cargo drives M2-like polarization and stromal remodeling	EV biogenesis inhibitors or RNA targeting	Preclinical	Blocks liver PMN in CRC models; redundancy limits efficacy	43, 44, 45, 46, 47, 48, 49
Target	TGF-β (dual with PD-L1)	Treg suppression + fibrosis programs	Bifunctional traps (e.g., bintrafusp alfa)	Phase I-II (terminated in some programs)	Variable efficacy; skin toxicity	89; NCT02517398
Target	IL-6/STAT3	Inflammatory signaling hub that supports MDSCs and Tregs and suppresses NK-cell activity	Anti-IL-6 or JAK/STAT inhibitors	Multiple oncology trials	Potential ICB synergy; infection risk	52, 53, 54
Biomarker	Exosomal miR-130b-3p/miR-21	Exosome-driven macrophage polarization and ECM/vascular remodeling	Plasma exosomes	∼75% sensitivity, ∼80% specificity (for CRC liver metastasis risk)	Early organotropism signal; needs validation	43,44
Biomarker	Serum 20-HETE	CYP4A lipid signaling activates STAT3 in TAMs	Serum	∼70% specificity (for breast cancer PMN priming)	Reflects metabolic switching; lifestyle confounding	18, 19, 20,88
Biomarker	Exosomal HSP90B1	Protein cargo promotes macrophage polarization and liver PMN initiation	Plasma exosomes	Prognostic association (advanced disease)	Possible hepatic tropism marker; redundancy possible	47
Biomarker	IL-6/GM-CSF profiles	Inflammatory hubs supporting suppressive myeloid/Treg programs	Serum	Moderate specificity (context-dependent)	Non-specific inflammation; confounders	52, 53, 54
Biomarker	Lipid-loaded monocytes (LD/FABP7 signatures)	Circulating myeloid lipid loading reflects immunometabolic priming	Blood flow cytometry	Exploratory (preclinical correlation)	Early warning potential; technical standardization needed	22, 23, 24, 25

Most listed clinical trials are not designed to specifically target the PMN; they primarily modulate macrophage/Treg pathways in established tumors and/or systemic immunity and are included here as PMN-relevant pathway interventions. PMN-directed trials will likely require validated niche biomarkers, risk-enriched enrollment, and metastasis-free/recurrence-free survival endpoints (e.g., perioperative or MRD settings). AHR: Aryl hydrocarbon receptor; Anti-PD-L1: Anti-programmed death-ligand 1; CCR8: C–C chemokine receptor type 8; CRC: Colorectal cancer; CSF1R: Colony-stimulating factor 1 receptor; CYP4A: Cytochrome P450 4A; ECM: Extracellular matrix; EV: Extracellular vesicle; FABP7: Fatty acid-binding protein 7; Fc: Fragment crystallizable; GM-CSF: Granulocyte-macrophage colony-stimulating factor; HETE: Hydroxyeicosatetraenoic acid; HSP90B1: Heat shock protein 90 beta family member 1; ICB: Immune checkpoint blockade; ICIs: Immune checkpoint inhibitors; IL-6: Interleukin-6; JAK: Janus kinase; KO: Knockout; LD: Lipid droplet; mAb: Monoclonal antibody; MDSC: Myeloid-derived suppressor cell; miR: MicroRNA; NK: Natural killer; PD-L1: Programmed death-ligand 1; PFS: Progression-free survival; PMN: Pre-metastatic niche; STAT3: Signal transducer and activator of transcription 3; STAT5: Signal transducer and activator of transcription 5; TAM: Tumor-associated macrophage; TGF-β: Transforming growth factor beta; Treg: Regulatory T cell.

### Macrophage targeting

Inhibiting CYP4A/20-HETE can decrease STAT3-driven PMN conditioning and metastasis in models, but systemic toxicity, such as hepatotoxicity, might limit clinical use.^18^, ^19^, ^20^ AHR inhibition reduces PD-L1-driven Treg expansion in lung PMNs; clinical development shows promise, though concerns about redundancy and toxicity persist.^10^^,^^59^^,^^60^ EV-targeted strategies, including cargo targeting or inhibiting EV biogenesis and uptake, can prevent macrophage reprogramming but encounter redundancy and delivery challenges.^39^, ^40^, ^41^, ^42^, ^43^ Myeloid engineering, like IL-12-genetically engineered myeloid cells (GEMys) and chimeric antigen receptor (CAR)-macrophages, enables targeted immune activation but requires further improvements in safety and scalability.^72^^,^^73^ Overall, effective approaches likely involve combining methods that block common pathways while restoring cytotoxic immunity to improve outcomes.^74^, ^75^, ^76^

### Treg targeting

Treg-directed approaches include CCR8 blockade/depletion, neural precursor cell expressed, developmentally downregulated 8 (NEDD8) inhibition, chemokine-axis blockade, and TGF-β/integrin targeting; however, these strategies carry risks such as autoimmunity and incomplete depletion, thereby supporting the need for biomarker-guided combinations.^56^, ^57^, ^58^^,^^77^, ^78^, ^79^

### Comparative weight of tumor-cell targeting vs pre-metastatic niche (PMN)-targeting therapies

Tumor-cell-directed therapies such as chemotherapy, targeted inhibitors, radiotherapy, and checkpoint blockade remain central to clinical treatment because they enable rapid reduction of tumor burden and measurable disease control, especially in cases with symptoms or high tumor load. However, their long-term effectiveness is often limited by factors such as intratumor heterogeneity, immune escape mechanisms, and acquired resistance.^80^

PMN-targeting strategies are best understood as “interception” approaches: they aim to prevent metastasis by disrupting immune-stromal interactions, such as macrophage-Treg circuits, vascular priming, ECM remodeling, and immune exclusion, before metastases become autonomous.^67^, ^68^, ^69^^,^^81^ Importantly, to date, there are few clinical trials explicitly designed to target the PMN as a distinct therapeutic entity. Instead, most clinical interventions target myeloid/Treg pathways in established tumors or modulate systemic immunity, and these pathways may indirectly affect influence PMN biology.

Because PMN biology is both time-sensitive and involves redundant pathways, the highly feasible clinical approach is to use combination strategies—tumor-cell targeting for immediate control, along with disruption of PMN-associated pathways to reduce recurrence and metastasis risk. This could also enhance immunotherapy effectiveness by alleviating suppressive conditioning.^4^^,^^10^^,^^81^ Accordingly, “PMN interception” should be evaluated using realistic trial designs (e.g., perioperative or minimal-residual-disease settings) with metastasis-free/recurrence-free survival and biomarker-defined niche modulation as endpoints, rather than assuming direct PMN-selective targeting by current trials.^4^^,^^7^^,^^81^

### Clinical trials, feasibility, and biomarkers for pre-metastatic niche (PMN) detection

Dedicated PMN-targeting interventional trials are currently rare, and there are virtually no trials explicitly designed to target the PMN as a distinct therapeutic compartment. Therefore, clinical evidence cited in this section should be interpreted as “PMN-relevant” (i.e., targeting macrophage/Treg or inflammatory pathways that may indirectly affect PMN conditioning), rather than PMN-specific intervention.

Although PMN-specific trials remain limited, multiple ongoing studies target pathways central to PMN biology, including myeloid reprogramming, Treg enrichment, and inflammatory hubs described earlier in the parts on macrophage reprogramming, macrophage–Treg crosstalk, and inflammatory signaling.^4^^,^^82^^,^^83^ These studies are primarily designed to modulate the tumor microenvironment at the primary site (and/or systemic immunity), and any PMN impact is inferred rather than directly tested.

For myeloid modulation, colony-stimulating factor 1 receptor (CSF-1R)-directed strategies have progressed into combination trials with checkpoint blockade; however, inflammatory toxicities and hepatotoxicity reported across programs highlight the importance of careful dosing, patient selection, and rational combinations.^83^^,^^84^ Notably, these CSF-1R studies are not designed to confirm PMN inhibition; instead, they evaluate tumor-site myeloid modulation with conventional clinical endpoints. Beyond macrophage-directed approaches, neutrophil plasticity and neutrophil extracellular trap formation (NETosis) represent additional PMN-relevant myeloid mechanisms that may contribute to tumor evolution, metastatic progression, and therapy resistance, emphasizing that myeloid-targeted interception may need to account for both macrophage and neutrophil programs.^85^

For Treg-focused strategies, CCR8 is attractive because it is enriched on tumor-resident suppressive Tregs; early clinical and translational studies support the feasibility of CCR8-directed depletion or antagonism, although durability may be limited by incomplete depletion and compensatory trafficking through parallel chemokines.^13^^,^^56^, ^57^, ^58^ However, these trials likewise do not operationalize the PMN as a measurable target site; thus, they should be viewed as pathway-directed trials with potential relevance to PMN biology, not PMN-selective interventions.

Engineered myeloid therapies demonstrate proof-of-concept for targeted immune rewiring: early studies of IL-12-GEMys and CAR-macrophage platforms show biological activity in solid tumors. However, challenges such as manufacturing variability, inflammatory toxicities, scalability, and identifying optimal combinations still exist.^72^^,^^73^ At present, these approaches are evaluated for tumor-site microenvironment remodeling rather than direct PMN interception.

Biomarkers are crucial for detecting active niche priming, stratifying high-risk patients, and tracking on-target pathway disruption. Potential liquid-biopsy signals include exosomal miRNAs associated with macrophage polarization and organotropism (e.g., *miR-130b-3p/miR-21*),^43^ lipid-metabolic mediators linked to TAM reprogramming (e.g., 20-HETE) 18, 19, 20, and exosomal protein cargo involved in PMN initiation (e.g., HSP90B1).^47^ Inflammatory cytokine profiles (e.g., IL-6/GM-CSF) can indicate suppressive conditioning but may lack specificity without additional context.^52^, ^53^, ^54^ Cellular metabolic indicators—such as lipid-loaded circulating monocytes (LD/FABP7 signatures)—are promising but still under investigation and require prospective validation.^22^, ^23^, ^24^, ^25^

Overall, achieving realistic clinical translation will depend on (i) validated biomarkers that define “PMN activation” in patients, (ii) enrolling patients based on risk factors (such as high-risk or minimal residual disease scenarios), and (iii) endpoints like metastasis-free or recurrence-free survival backed by biomarker evidence of niche modulation. Until these tools and approaches are fully developed, current trials should be considered mechanistically relevant to the PMN rather than specifically targeting it.^4^^,^^6^^,^^81^

## Future directions

Clinical translation necessitates organ-specific PMN mapping, validated early biomarkers, and therapies that target interconnected immune-stromal circuits. Spatial atlases combining single-cell and multiplex imaging can facilitate organ-specific interventions, while longitudinal liquid biopsies—such as EV ncRNAs, lipid metabolites, and cytokines—may help stratify risk and track on-target effects. Future research should focus on developing rational combination therapies that target both shared hubs and organ-specific drivers, testing them within optimal clinical windows (perioperative, high-risk, minimal residual disease) across diverse populations.^4^^,^^6^^,^^81^

## Conclusion

The PMN is an actively tumor-engineered ecosystem that facilitates metastasis through immune suppression, vascular priming, and stromal remodeling. TAMs and Tregs create key, self-reinforcing circuits that inhibit NK/CD8^+^ immunity, drive ECM and vascular changes, and influence DTC fate. Targeting common hubs such as STAT3, NF-κB, and AHR, as well as EV-mediated programming, lipid metabolic rewiring (e.g., CYP4A/20-HETE; FABP7/lipid droplet programs), and Treg recruitment/function (e.g., CCR8 axes), presents opportunities for preventing metastasis. Metabolic stress pathways, like exosomal TRAIL under glucose restriction, further reveal connections between tumor metabolism and immune remodeling at distant sites.^50^, ^51^, ^52^ Appropriately designed combination therapies, guided by biomarkers and organ-specific factors, may enhance treatment durability and lower metastatic risk.

## Authors contribution

Qamar Abuhassan: Conceptualization, manuscript design, writing - original draft preparation, and figure conceptualization/visualization; Ghaleb Oriquat: Writing–review and editing, literature survey, and critical intellectual input; Waleed K. Abdulsahib: Conceptualization, supervision, writing, review and editing, and final approval of the manuscript; R. Roopashree: Literature search, data curation, writing–review and editing; Priya Priyadarshini Nayak: Clinical perspective integration, literature validation, writing–review and editing; J. Bethanney Janney: Writing–review and editing, technical refinement, and validation; Vipasha Sharma: Data interpretation, schematic refinement, and writing–review and editing; Xushvakt Umedov: Writing–review and editing, validation, and consistency checking across sections. All authors have read and approved the final manuscript.

## Ethics statement

None.

## Data availability statement

The datasets used in the current study are available from the corresponding author on reasonable request.

## Declaration of generative AI and AI-assisted Technologies in the writing process

During the preparation of this work, the authors used Grok and ChatGPT to improve the writing process and enhance the manuscript’s readability and language. After using these tools, the authors reviewed and edited the content as needed and take full responsibility for the content of the published article.

## Funding

None.

## Conflict of interest

The authors declare that they have no known competing financial interests or personal relationships that could have appeared to influence the work reported in this paper.
